# Consulting patients in setting priorities in Myalgic Encephalomyelitis (M.E.) research: findings from a national on-line survey

**DOI:** 10.1186/s40900-015-0011-x

**Published:** 2015-09-28

**Authors:** Nicola Childs, Lisa Robinson, Sonya Chowdhury, Clare Ogden, Julia L. Newton

**Affiliations:** 1 Clinical Academic Office, The Medical School, Newcastle University, Newcastle, NE2 4HH, UK; 2 Action for M.E, 42 Temple Street, Keynsham, BS31 1EH UK; 3 Fatigue CRESTA Clinic, Newcastle Hospitals NHS Foundation Trust, Newcastle, UK

## Abstract

Myalgic encephalitis (M.E.) is a common condition, the cause of which is not known and there are no treatments available. In this study the national patient support group Action for M.E. sought the opinions of their members via an online survey as to what they felt should be future priorities for M.E. research.

Respondents were asked what they considered first, second and third research priorities to be from a list of 13 pre-defined options. Individuals were invited to provide additional free text comments about Action for M.E.’s research priorities in general.

Of the 1144 respondents: 822 had M.E.; 94 were a supporting a member of Action for M.E. ; 66 were carers for someone with M.E.; 26 were professionals with an interest in M.E.; 136 had a family member or colleague with M.E. Individuals selected more than one category as applicable.

The top five research priorities identified were: disease processes to achieve a better understanding of the causes of M.E.; more effective treatments; faster and more accurate diagnosis; clinical course of M.E.; outcomes and natural history; and severely affected patients. Least popular priorities were: sleep; economic research towards identifying the cost of ME; and psychological aspects. Much of the free text comments emphasised the importance of funding biomedical research into disease processes to achieve a better understanding of the causes of M.E. Three themes were identified in relation to this topic: accurate diagnosis and awareness; risk factors and causes; drug development and curative therapies.

In conclusion; individuals affected by M.E. have clear views regarding priorities for research investment. These have informed Action for M.E.’s ongoing research strategy and ultimately will inform national and international research priorities.

## Abstract (fr)

**Background:**

The aim of this work was to involve patients in setting future priorities for myalgic encephalomyelitis (M.E.) research.

**Methods:**

A national on-line survey was developed collecting structured and unstructured data. Respondents were asked what they considered Action for M.E.’s first, second and third research priorities to be from a list of 13 pre-defined options. Individuals were also invited to provide any additional free text comments about Action for M.E.’s research priorities in general. A total of 1144 individuals completed the on-line survey. Respondents were asked to indicate if: they had M.E. (n = 822; 90.4 %); were a supporting a member of Action for M.E. (n = 94; 10.3 %); carer for someone with M.E. (n = 66;7.3 %), professional with an interest in M.E. (n = 26;2.9 %); or had a family member or colleague with M.E. (n = 136;15 %). Individuals were able to select more than one category as applicable.

**Results:**

The top five research priorities identified by the respondents were: disease processes to achieve a better understanding of the underlying pathology of M.E.; more effective treatments; faster and more accurate diagnosis; clinical course of M.E.; outcomes and prognosis; and severely affected patients. The lower research priorities identified were: sleep; economic research towards identifying the cost of ME for individuals and society; and psychological aspects. Much of the unstructured data provided by respondents emphasised the importance of funding biomedical research into disease processes to achieve a better understanding of the underlying pathology of M.E. Three themes were identified in relation to this topic: accurate diagnosis and awareness; risk factors and causes; drug development and curative therapies.

**Conclusions:**

Individuals affected by M.E. have clear views regarding the most important priorities for research investment. These tended to focus on disease processes to achieve a better understanding of the underlying pathology of M.E. and have been used to inform Action for M.E.’s ongoing research strategy.

## Article summary

### Strengths

- Results of a survey performed from the largest National Patient Support group for ME – Action for M.E.
- Research priorities derived and defined to inform funders and policy makers and ultimately to act as a driver for increased research funding in M.E.

### Limitation

- Survey methodology deployed via social media which may have influenced those able to access the survey.
- Selective group of participants who volunteered their participation.

## Background

The importance of involving patients in the development and monitoring of health services and in the conduct of health services research is widely recognised throughout the developed world [1–6]. The UK government’s commitment to patient involvement is evident through the publication of documents such as Our Health, Our Care, Our Say [7] and Equality and Excellence: Liberating the NHS [8]. Similarly, the National Institute for Health Research (NIHR) actively encourages patient involvement in NHS, public health and social care research through its advisory group INVOLVE [9]. The advantages of involving patients in health services research are well documented [10–13], and guidelines have been produced recommending the inclusion of patients at every stage of the research process [14]. Involving patients in setting the research agenda is particularly important as their priorities may differ from current research practice, and are more likely to reflect the interests of the NHS, public health and social care services [15]. In keeping with the core democratic principles of active citizenship, accountability and transparency, the legitimacy and sustainability of investment decisions made by research funding bodies will be increasingly influenced by how well they reflect the underlying values of the general public [16].

Despite examples of good practice in areas such as COPD and asthma [17], cancer [18] and chronic kidney disease [19], consulting patients when determining research priorities remains a developing area of study [15]. Myalgic Encephalomyelitis (M.E.) is a complex multi-system disorder that affects an estimated 250,000 men, women and children in the UK [20]. Symptoms include post-exertional malaise, fatigue, sleep disturbance, widespread muscle and joint pain, and difficulties with memory and concentration [21]. However, each patient experiences their own personal combination of symptoms, posing significant challenges for healthcare professionals in relation to diagnosis and management [22]. Although there have been attempts to gather the views of those affected by M.E. regarding research that individuals would be willing to participant in [23, 24], to date, there have been no comprehensive attempts to involve patients in determining the strategic direction of M.E. research at a national level. Given the lack of understanding regarding the disease mechanisms underpinning M.E .and the uncertainties associated with diagnosis and management, it would seem even more critical that the views of individuals affected by M.E. are listened to and acted upon when determining future research priorities. Further complexity arises from differing nomenclature with the terms M.E. and chronic fatigue syndrome (CFS) sometimes being used interchangeably.

In response to the paucity of literature surrounding the research agendas of individuals with M.E. the UK-based charity Action for M.E. initiated the first national on-line survey to involve people affected by M.E. in identifying priorities for future research investment. This paper will present the key findings from this work.

## Methods

**Fig. 1:**
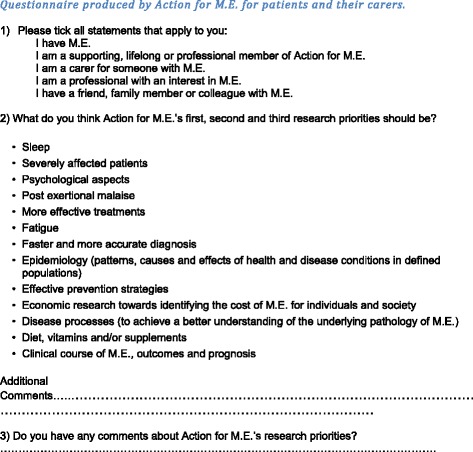
The on-line survey

A three question survey (Fig. 1) was used to gather structured and unstructured data to inform views regarding national funding priorities for M.E. research. Participants were asked what they considered Action for M.E.’s first, second and third research priorities should be from a list of 13 pre-defined options (sleep; severely affected patients; psychological aspects; post-exertional malaise; more effective treatments; fatigue; faster and more accurate diagnosis; epidemiology patterns, causes and effects of health and diseases conditions in defined populations; effective prevention strategies; economic research towards identifying the cost of M.E. for individuals and society; disease processes to achieve a better understanding of the underlying pathology of ME; diet, vitamins and/or supplements; and clinical course of M.E., outcomes and prognosis). These categories were defined collaboratively by the Action for M.E. Patient Reference Group and Research Panel. A comments box was available for respondents to add free text responses. Individuals were also invited to provide any additional free text comments about Action for M.E.’s research priorities in general.

### Participant recruitment

Respondents were asked to indicate if: they had M.E.; were a supporting a member of Action for M.E.; a carer for someone with M.E., a professional with an interest in M.E.; or had a family member or colleague with M.E. Individuals were able to select more than one category as applicable. The survey was designed to be inclusive to anyone wishing to participate and was available on-line via Action for M.E.’s website, Facebook and Twitter pages for a 4 week period between 23.05.13 and 23.06.13.

### Data analysis

The structured data were collated, categorised and subjected to graphical representation. All data was collated anonymously, no personal details were collected.

The unstructured data were subjected to framework analysis [25]. This approach consisted of a series of five distinct, yet highly interconnected, stages (familiarisation; identifying a thematic framework; indexing; charting; mapping and interpretation), and enabled the themes to be informed by the structured data as well as the free text provided by respondents.

## Results

### Participants

**Fig. 2:**
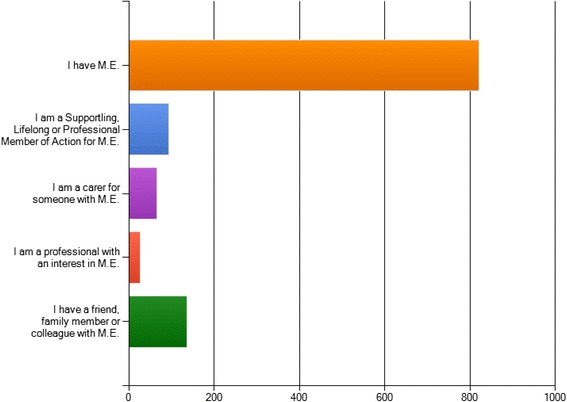
Details of the numbers of respondants

A total of 1144 participants completed the on-line survey (Fig. 2). The majority of respondents indicated that they had M.E. (n = 822). However, responses were also received from individuals who had a family member, friend or colleague with M.E. (n = 136) or were caring for someone with M.E. (n = 66). Individuals with a professional interest in M.E. also completed the online survey (n = 26), and a proportion indicated that they were a supporting lifelong or professional member of Action for M.E. (n = 94).

### Top five research priorities described by Action for M.E. survey respondents

**Fig. 3:**
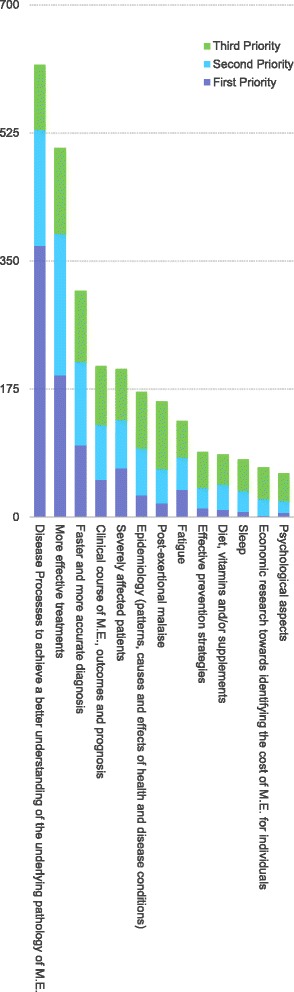
The priorities identified by respondants to the on-line survey

The top five areas identified as a first, second or third research priority were: disease processes to achieve a better understanding of the underlying pathology of M.E. (total rating count = 618); more effective treatments (total rating count = 505); faster and more accurate diagnosis (total rating count = 309); clinical course of M.E., outcomes and prognosis (total rating count = 207); and severely affected patients (total rating count = 203). The least popular research priorities identified were: sleep (total ratings count = 79); economic research towards identifying the cost of M.E. for individuals and society (total ratings count = 68); and psychological aspects (total rating count 60). (Figure 3 and further details are available from http://www.actionforme.org.uk/get-informed/news/our-news/archived-news/our-news/2013/research-priorities-our-survey-results).

### Key themes from the unstructured responses to the research priorities survey

Much of the unstructured data provided by respondents emphasised the importance of funding biomedical research into disease processes to achieve a better understanding of the underlying pathology of M.E. Three themes were identified in relation to this topic, and these will provide the basis for the presentation of the unstructured responses.

- Theme 1: Accurate diagnosis and awareness
- Theme 2: Risk factors and causes
- Theme 3: Drug development and curative therapies

#### Theme 1: accurate diagnosis and awareness

Many of the respondents felt their condition was often trivialised and requested that a clear distinction be made between M.E. and Chronic Fatigue Syndrome (CFS).

*A “Define a more accurate name for the illness”*

*B “Chronic fatigue is not M.E. and has trivialised M.E., making the dangers in the condition misunderstood”*

*C “Change the name of M.E. – ‘chronic fatigue’ dropped and M.E. as M.E. given the neurological symptoms, effects and underlying pathology of the condition”*

It was suggested that the identification of a diagnostic test/biomarker would promote accurate and timely differential diagnosis.

*“Identify a marker for M.E.”*

*“A diagnostic test to separate it from chronic fatigue”*

Several of the respondents described a general lack of sensitivity on the part of health and social care professionals in their interactions with individuals with M.E.

*D “A law to stop abuse from doctors and social services”*

*E “GPs who believe you when you visit”*

It was suggested that education and training should be provided as a matter of urgency to raise awareness of M.E. amongst health and social care professionals.

*F “As some doctors don’t believe M.E. exists, there should be compulsory courses for GPs on the symptoms and their effects on patients”*

Respondents provided specific details of their own personal disease history and experiences of being diagnosed with M.E. Many indicated that they had experienced long delays in receiving a definitive diagnosis and that this had affected their ability to commence treatment in a timely manner.

*G “Ensuring that once a patient is referred to their local CFS/M.E .service that appropriate advice is supplied to stop serious deterioration while waiting for help”*

*H “I have had M.E. for 28 years now. I have no-one who I go to see, either a doctor or a specialist”*

*I “Diagnostic help. 1989 M.E. started, 1992/3 diagnosed, 70 next year, still with M.E.”*

Several respondents indicated that a lack of awareness of M.E. and delay in diagnosis had impacted on their ability to receive financial support to help manage their condition and remain independent in activities of daily living.

*J “Ensuring that people receive benefits so that they can survive”*

*K “Financial help to pay for help in the home, as those with no-one at all to help with the day to day running of their home, and this is not a luxury – it’s to obtain a reasonable way of living”*

#### Theme 2: risk factors and causes of M.E

It was suggested that research should focus on understanding the risk factors and underlying causes of M.E. Again, respondents indicated that a distinction should be made between M.E. and CFS when prioritising future research funding.

*L “It’s obvious to me that the priority is research into the causes of this dreadful illness”*

*M “Research should concentrate on the biology of severe Myalgic Encephalomyelitis and not the psychology of mild Chronic Fatigue Syndrome”*

Respondents demonstrated their awareness of, and support for, biomedical research into a potential immunological/viral association with M.E.

*“Immune deficits and viruses”*

*“Testing for viruses, vitamin deficiencies and food intolerances”*

*“Gut flora”*

*“Effects of ageing”*

Many respondents emphasised the importance of developing an enhanced understanding of the disease processes underlying their condition.

*“Mainly interested in a proper understanding of the underlying disease spectrum”*

Respondents also demonstrated their awareness of, and support for, biomedical research into the disease processes underpinning M.E. at a molecular level.

*N “Understanding the effect of malfunction in the mitochondria organ in the cell, where all of the body’s energy is created”*

*“Cellular activity within muscles, dysfunctioning methylation cycle”*

#### Theme 3: drug development and curative therapies

Several of the respondents indicated that research should focus on developing effective interventions for M.E., with a particular emphasis on curative therapies.

*“Research into effective treatments and hopefully a cure”*

*“Treatment or a cure is a priority”*

It was suggested that the focus of treatment should move away from psychological therapies, which were felt to have limited benefit for individuals with M.E.

*O “I would also like to see much less emphasis on any psychological intervention or therapies. Of course people become depressed – but it’s the illness first and any psychological factors follow”*

*P “Action for M.E.’s research priorities need to be focused on proper biomedical research and should not be allowed to drift back to funding research linked to the biopsychosocial model proposed by certain UK psychiatrists. This is a dead-end and total waste of precious research funds”*

*“No CBT or GET as I found it made me ten times worse”*

Respondents felt that funding should be invested in clinical trials to develop effective drug management for M.E.

*“Immune system studies including treatments with immune modulators such as Rituximab”*

*“Rituximab, Ampligen and Oxygen Therapy - do they help people with M.E.”*

## Discussion

This survey is unique in seeking the views of patients in determining the strategic direction of M.E. research at a national level. Contrary to common perceptions [15], we have demonstrated that individuals affected by M.E. are able to engage with a broad range of issues relating to science, medicine, and healthcare delivery and can identify and agree on research priorities. The top five research priorities identified by the respondents of this on-line survey were: disease processes to achieve a better understanding of the underlying pathology of M.E.; more effective treatments; faster and more accurate diagnosis; clinical course of M.E.; outcomes and prognosis; and severely affected patients. The least popular research priorities identified were: sleep; economic research towards identifying the cost of M.E. for individuals and society; and psychological aspects. The historical research effort in M.E. does not obviously match with the prioritisation seen in this survey from consumers, highlighting how in the past the research community’s priorities may not have aligned with those of the patients or consumers. To date, there have not been national research priorities and this current work will ensure that the patients’ priorities must now be considered.

A particular strength of this work was the inclusion of free text responses, which enabled individuals affected by M.E. to articulate their views and actively influence the strategic direction of future research funding in the UK. In keeping with the research priorities set out by a collaborative clinical sub-group [26], respondents emphasised the importance of investing in research to achieve a better understanding of the disease processes underpinning M.E. Many expressed concerns at the trivialisation of M.E. by health and social care professionals, and the labelling of patients with M.E. as suffering from a psychiatric, psychological or somatic symptom disorder. Respondents demonstrated awareness of, and support for, biomedical research into the pathogenesis of M.E. at a molecular or genetic level. It was suggested that the identification of protein biomarkers would provide a clear distinction between M.E. and CFS, facilitating accurate diagnosis and timely access to tailored interventions with a particular emphasis on drug development and curative therapies. A high proportion of respondents provided their personal contact details with a view to being involved in future research prioritising disease mechanisms, a diagnostic test and specific therapies.

Given the paucity of literature surrounding the research priorities of individuals affected by M.E., it was necessary to undertake an exploratory scoping exercise. However, this survey has a number of limitations. Despite the large number of responses and willingness to participate in future research, it is important to acknowledge that the individuals with M.E. completing this survey were a self-selecting group for which no demographic information was available. It is, therefore, not possible to say whether the views presented in this paper are representative of the 250,000 people currently estimated to be living with M.E. in the UK. The survey was only available on-line via Action for M.E.’s website, Facebook and Twitter pages. For this reason, certain groups, such as the severely affected or those with lower computer literacy levels, may have been excluded from providing their opinions. Although research into the pathophysiology of severely affected individuals was identified as one of the top five research priorities, it is not possible to say whether this patient group were adequately represented in this survey. Carers for someone with M.E. or individuals with a family member of colleague with M.E. were also under-represented, as were professionals with an interest in M.E. although there were no obvious differences in research priorities between patients, carers and family members. Only six healthcare professionals provided unstructured data to support their first, second and third research priorities, limiting the potential for meaningful comparison between groups.

Although still a developing field of study, healthcare organisations throughout the developed world are increasingly involving patients in service delivery and policy decisions, including guideline and quality indicator development, programme development and evaluation, quality improvement and funding priorities. The findings from the on-line survey have been used to inform and influence the statement of intent underpinning the largest national patient charity Action for M.E.’s Research Strategy 2014–2016 [27] which seeks to provide personalised medicine for patients with M.E. through the prioritisation of four key research areas: cause and prevention; diagnosis; targeted treatments; and severely affected individuals. The rise in the number of people living with chronic disease, such as M.E., highlights the importance of productive interactions between funding bodies, research charities, patients and healthcare professionals. In the field of M.E. over recent years the coming together of the five largest national patient charities together with researchers and clinicians to form the UK CFS/ME Research Collaborative has provided the framework to consider and provide focus to address the research priorities outlined in the survey. Future work should focus on promoting further transparency and clarity in the reporting of achievements against these set targets.

The study has some limitations. The participants are clearly a self selected group who are members of the largest national patient support charity for M.E. It is therefore important to consider their responses in the context of this self selection and recognise that the respondents are not randomly selected. It is also not possible to determine the absolute reach of the survey, as it was deployed via the social media outlets and website for the charity for a very defined period of time. It is therefore important that more rigorous research is performed to explore in more detail the research priorities for those with M.E. and to specifically establish service user led priorities. We would suggest that this survey with all the associated limitations of survey work has highlighted an area where those with M.E. feel that they wish to influence policy. This is underlined by not only the numbers of respondants but also by the richness of the free text included with the quantitative responses. We believe that this is a strong platform upon which to further enhance our understanding of the priorities of those affected by M.E.

## Conclusions

This work illustrates the importance of involving individuals affected by M.E. in the identification of strategic priorities for future research funding. As the beneficiaries of discoveries and developments in health services research, it is particularly important that the views of patients are listened to and acted upon. We have demonstrated that individuals affected by M.E. have clear views regarding the important priorities for research investment. These tended to focus on disease processes to achieve a better understanding of the underlying pathology of M.E. and would appear to be closely aligned with those of the scientific research community.

### Data sharing

The original data from the survey is available from Action for M.E. in an excel spreadsheet.
